# Rationale and feasibility of a combined rapid assessment of avoidable blindness and hearing loss protocol

**DOI:** 10.1371/journal.pone.0229008

**Published:** 2020-02-13

**Authors:** Tess Bright, Ian McCormick, Mwanaisha Phiri, Wakisa Mulwafu, Matthew Burton, Sarah Polack, Islay Mactaggart, Jennifer L. Y. Yip, De Wet Swanepoel, Hannah Kuper

**Affiliations:** 1 International Centre for Evidence in Disability, London School of Hygiene & Tropical Medicine, Keppel St, London, United Kingdom; 2 International Centre for Eye Health, London School of Hygiene & Tropical Medicine, Keppel St, London, United Kingdom; 3 Audiology Department, Queen Elizabeth Central Hospital, Blantyre, Malawi; 4 Department of Surgery, College of Medicine, Blantyre, Malawi; 5 Department of Speech-Language Pathology and Audiology, University of Pretoria, Pretoria, Gauteng, South Africa; LV Prasad Eye Institute, INDIA

## Abstract

**Purpose:**

This study has two main objectives: 1) to assess the value of combining the rapid assessment of avoidable blindness (RAAB) and the recently developed rapid assessment of hearing loss (RAHL) based on existing population-based data from Cameroon andIndia; 2) to test the feasibility of a combined RAAB-RAHL protocol.

**Methods:**

A secondary data analysis of population-based disability surveys in India and Cameroon (in 2013–2014) was conducted, focussing on people aged 50+. Hearing impairment (HI) was defined as pure tone average of ≥41dB (better ear).Visual impairment (VI) was defined as presenting visual acuity of <6/18 (better eye). The relationship between HI and VI was examined. The feasibility of a combined RAAB-RAHL survey was assessed within a RAHL conducted among adults aged 50+ in Malawi in 2018. Outcomes included: time taken, costs, number of people examined in a day, and qualitative feedback from participants and field teams.

**Results:**

The prevalence of combined VI and HI among people aged 50+ was 4.4% (95% confidence interval (CI) 3.0, 6.4) in India and 4.8% (95%CI 3.0, 8.0) in Cameroon. Among participants with VI, approximately a third in India (29.3%) and Cameroon (35.1%) also had HI. A quarter of participants in India (25.4%) and Cameroon (26.9%) who had HI also had VI. In Malawi, the total time taken to complete both RAAB and RAHL assessments was approximately 27 minutes per participant. It was feasible to complete 30 participants per day for a team of four people. The estimated cost of a combined RAAB-RAHL approach in comparison to two separate impairment surveys is up to 37% less depending on the method of combination.

**Conclusion:**

The substantial overlap between VI and HI supports a combined rapid survey of the two impairments. The pilot study of a combined RAAB-RAHL survey demonstrates feasibility and lower cost compared to conducting two standalone impairment surveys. A combined RAAB-RAHL approach could maximize limited resources to increase prevalence data for both vision and hearing impairment.

## Introduction

Globally, more than 250 million people have visual impairment (VI) and over 450 million have hearing impairment (HI).[1–3] The prevalence of VI and HI tends to be higher in low- and middle-income countries (LMICs).[1, 4] Over the next 30 years, with ageing and global population growth, the number of people affected by both impairments is expected to rise–to 700 million (1 in 12 people) with VI and 900 million (1 in 10 people) with HI.[1, 3] Reliable data on needs and unmet needs for eye and ear care services are needed for planning purposes.

The Rapid Assessment of Avoidable Blindness (RAAB), has been a successful approach to collecting population-based data on the prevalence and causes of blindness and VI in a low-cost and rapid manner.[5] More than 300 surveys have been undertaken using this method, the majority of these in LMICs.[6] RAABs are used to support public health planning and implementation, thereby contributing substantially to global estimates of blindness.[6] The survey is considered “rapid” for several reasons. First, the target population is people aged 50 years and above, given that the prevalence of blindness is highest in this older population. Evidence suggests that over 80% of blindness is experienced by those aged 50+, and that the distribution of the main causes of blindness in this age group are comparable to the all-age population.[7] Therefore, focussing on this age group substantially reduces the sample size required to accurately estimate the prevalence, yet still generates data that can be used to plan eye care services for the total population. Secondly, a simplified clinical protocol, using low-cost equipment, is used to test vision and examine eyes. This allows testing to be done relatively quickly in the participant’s home. Finally, automated data analysis and reporting is possible through bespoke and freely available software.[6] Given the standardised analysis, the results from RAAB surveys can be compared over time and across settings.

In contrast to VI, population-based data on HI are limited, particularly in LMICs. For example, Global Burden of Disease (GBD) estimates are based on less than 50 surveys of HI, many of which are outdated.[4] Both HI and VI prevalence increases substantially with age.[4] Analysis of previous population-based survey in an all-age population has demonstrated that the majority (>70%) of HI cases are experienced by people aged 50+.[8] Compared to vision, causes of HI are more difficult to determine in both clinical and survey settings.[9] However, data have shown (similarly to vision) that the distribution of the causes amongst the 50+ population is comparable to the total population.[8] Given these findings, a Rapid Assessment of Hearing Loss (RAHL) survey protocol has been developed, based on the RAAB, taking a pragmatic approach to collecting population-data on HI quickly and at relatively low cost in order to inform service planning.[8] The RAHL has potential, as demonstrated with RAAB, to significantly increase available HI prevalence data.

Combining the RAAB and RAHL surveys could maximise the value of limited resources available for conducting population-based surveys, particularly in LMICs. The rationale for this approach is two-fold. Firstly, both surveys adopt the same sampling methodology (two stage cluster sampling: probability proportionate to size, followed by compact segment sampling). Secondly, they target the same population (aged 50+) given the heightened risk for both VI and HI in this age group. Sensory impairment, which includes HI and VI, is a leading contributor to the GBD, particularly for older populations.[10] Studies have also shown an association between the two impairments amongst older populations.[11, 12] In Australia, for example, the prevalence of combined VI and HI among people aged 55 years and older was 6.0%, HI was present in 65.2% of people with VI and participants with VI were 1.5 times more likely to have HI.[12]. Both hearing and vision impairment are associated independently with increases in depression, falls, mortality and decreased quality of life.[11] Further, people with both impairments have been reported as having poorer health and quality of life than people without impairments, and those with a single sensory impairment.[11–13] There is little data on the associations between VI and HI from LMICs. Greater efforts are needed to ensure that the cumulative effects of sensory impairments amongst this population can be addressed, and functioning can be maximised. Collecting population-based data on both impairments, therefore, has the potential to allow comprehensive service planning for sensory impairment. This is particularly important in the context of global ageing populations.[14] Another rationale for a combined survey protocol is that in practice, a large proportion of the time spent in RAAB surveys is used to identify eligible individuals. Once identified, performing additional clinical assessments, including assessing hearing function and examining ears, should be relatively straightforward. However, the feasibility of a combined approach needs to be tested.

This study has two main objectives. Firstly, to consider the rationale for combining the RAAB and RAHL based on existing population-based data from two LMICs. Secondly, to test the feasibility of a combined protocol in Ntcheu district, Malawi.

## Materials and methods

### Rationale for combining RAAB and RAHL

We conducted secondary analysis of datasets to explore the relationship between VI and HI in two LMICs and the potential value of combined rapid surveys.

#### Data collection

In 2013–2014, all-age population-based surveys of disability were undertaken in one district each in India (Mahbubnagar District) and Cameroon (Fundong Health District).[15, 16] These surveys measured VI and HI using both self-reported and clinical tools. Sample size calculations resulted in a target sample size of 4056 participants of all-ages in 51 clusters of 80 people. Probability proportionate to size sampling was used to select clusters, using the most recent census as a sampling frame. Within clusters, households were selected using compact segment sampling. All individuals aged 50+ living in the selected households were screened for hearing and visual impairments:

#### Hearing impairment

Two stage hearing screening was conducted. Otoacoustic emissions (OAE) testing was conducted first (Otodynamics), followed by pure-tone audiometry (Interacoustics screening audiometer AS608) for participants who failed OAE in both ears. Two types of OAEs were utilised, distortion product (DPOAE) and transient evoked (TEOAE). The collection parameters for DPOAEs included frequencies of 2, 3, 4, and 6kHz. TEOAEs screened the following frequencies: 1, 1.5, 2, 3, and 4 kHz. The pass criteria for both instruments was set to signal to noise ratio of 6 dB and a minimum signal of >−5 dB SPL in at least three frequencies

Testing was conducted by an audiologist in a central location (e.g. community building) in each of the selected clusters.[16, 17] Hearing thresholds were obtained at frequencies 0.5, 1, 2, and 4kHz in each ear. Cases were defined as those with pure-tone average of thresholds obtained of ≥41dBHL in the better hearing ear.[18] Identified cases were examined by an experienced clinician who specified the main probable cause of HI based on otoscopic examination and clinical history.

#### Visual impairment

The RAAB6 survey protocol was followed to measure VI.[19] Presenting (with spectacle correction if available) distance visual acuity (VA) was measured in each eye using a tumbling “E” chart with 6/12 (India only), 6/18 and 6/60 optotypes. Where presenting VA in an eye was worse than 6/12 (India) or 6/18 (Cameroon), the measurement was repeated with a pinhole to assess for refractive error. Lens examination was undertaken in all participants using a torch and direct ophthalmoscope by an ophthalmic nurse or assistant. The main cause of reduced VA (<6/18) was assigned from the pre-existing RAAB list of ocular pathologies, both at the level of the eye and the individual.

#### Case definitions

The definitions used for sensory impairments were:

- **VI:** Presenting VA <6/18 in the better eye [20]
- **HI**: Pure tone average (of thresholds at 0.5, 1, 2, 4kHz) in the better ear of ≥41dB; or fail OAE in both ears [21]

These align with the WHO definitions used to calculate global statistics for moderate or greater HI and VI.

#### Analysis

Data were analysed in Stata version 15.0, using the “svy command” to account for the cluster survey design. The prevalence and 95% confidence intervals (CI) of hearing, visual, and combined sensory impairment among people aged 50+ were estimated. Logistic regression was used to determine the odds of HI amongst participants with VI, and vice versa, in each country. Age and sex were adjusted for as *a priori* confounders.

### Pilot study of combined RAAB and RAHL

#### Study setting

This study was set in Ntcheu district, Central Malawi. Ntcheu district has a population of approximately 650,000 people of whom 9% are 50+, across 834 villages. The district is predominantly rural, covering an area of over 3000 square kilometres, and a population density of 108 people per square kilometre. The district is served by one secondary level district hospital, which provides basic ENT services.

#### Study design and sampling

A population-based survey of HI in people aged 50+ (i.e. a RAHL) was conducted during November and December 2018.[8] Probability proportionate to size sampling was used to select clusters, using the most recent census (2008) as a sampling frame. Within clusters, households were selected using compact segment sampling and all eligible people aged 50+ were included in the survey.[22] The cluster size was based on pilot testing of the methodology in Malawi, which found that the number of people that can be feasibly assessed in one day is 30. This is smaller than the cluster size used in RAAB of 50 because the examinations take longer to complete.

A feasibility study of a RAHL survey in combination with a RAAB survey was nested within the population based study. Feasibility studies are used to answer the question “can this study be done?”.[23] The combined RAAB-RAHL protocol was nested within the first two weeks of the survey in nine out of 38 clusters (target of 270 people). This represents nearly a quarter (24% of included clusters) and was deemed to be adequate for determining feasibility.[24]

#### Teams

The RAHL survey was conducted by two teams, each comprising four people:

1. An enumerator (nurse) who identified eligible individuals and completed a short sociodemographic questionnaire;
2. Two hearing testers (audiology officer or trained nurse) who completed the hearing test [15];
3. An ENT Clinical Officer who examined ears and obtained a clinical history [15].

In nine clusters of the survey an Ophthalmic Clinical Officer (OCO) joined one of the teams to conduct visual acuity testing and eye examination. In Malawi, a clinical officer is a mid-cadre health worker with some specialist training.

#### Data collection

All data were collected on mobile-based questionnaires, using Open Data Kit (ODK) Collect in the participant’s home. Hearing was assessed using hearTest, which is a validated smartphone-based automated pure tone audiometry system.[25, 26] hearTest was coupled with circumaural noise attenuating headphones (Sennheiser HD280).[27] Testing was conducted in a quiet room in the participant’s home.Thresholds were assessed at 0.5, 1, 2, 4kHz. As in India and Cameroon, cases were defined as those with pure-tone average of thresholds obtained of ≥41dBHL in the better hearing ear. All participants had their ears examined by an ENT clinical officer, and likely causes assigned.

For vision, the RAAB6 survey protocol was used with some minor modifications as a result of combining it with RAHL. Firstly, there was only one rather than the typical two RAAB team members and secondly cluster sizes of 30 instead of 50 were used.[22] A full vision assessment using the Peek Acuity mobile application at 3 metres was trialled in the first three clusters.[28] However, the approach of testing all eyes down to 6/6 was time-consuming and, thereafter, the standard RAAB6 Tumbling E screening test (to 6/12) was employed instead.[5, 19, 22] The most recent iteration of the RAAB survey methodology includes a screening version of the Peek Acuity vision test for mobile devices which tests for vision at the 6/12 threshold level, not available at the time of the pilot. Participants with VI, underwent an undilated eye examination using an Arclight to assess cause.[29] The RAHL exam was conducted first, followed by the RAAB exam.

#### Feasibility assessment

Data on the following outcomes were recorded to assess the feasibility of the combined survey approach: time taken to complete the RAAB component of the survey, time taken to complete the RAHL component of the survey, and associated costs. Duration variables were created based on the difference between the start and end time of each questionnaire (i.e. RAHL and RAAB questionnaires), and in total across questionnaires. VA tests conducted with Peek (n = 45/188) were excluded from the analysis given it was deemed too time consuming, and not usually included in the RAAB protocol. Time taken to obtain consent was recorded through ad hoc field observations (n = 16) throughout the first two weeks of data collection. In addition, we sought informal verbal feedback from the ENT, OCO and survey coordinator about: field work logistics, clinical experience of examiners, and the perceived acceptability of the combined methodology for participants.

Cost data were extracted from financial reports. The actual costs from Malawi in Great British Pounds (GBP) for the RAHL survey and estimated typical costs of a RAAB survey ($40,000 USD in 2013, inflated to 2018 costs, converted to GBP = £33,000) [30] were used to project the costs for a combined survey. The equipment costs were estimated based on previous RAAB and RAHL surveys, as for this survey all equipment was borrowed. The sample size for RAAB (typically between 3000–5000 people) is typically larger than RAHL (1000–2000 people). This is because RAAB powers the sample size on the expected prevalence of blindness, and the RAHL is powered for moderate or greater HI. Therefore, we estimated costs using different potential scenarios for combining the two methods, including:

1. RAAB and RAHL completed separately
2. RAAB and RAHL combined, sample size powered to estimate the prevalence of moderate VI and blindness
3. RAAB in all clusters; and RAHL in 1/3 clusters (RAHL requires approximately one third of the participants than RAAB, depending on the expected prevalence).
4. RAHL with VI survey; powered to estimate prevalence of moderate or worse VI (i.e. modified RAAB as not powered to estimate prevalence of blindness) and moderate or greater HI

#### Data analysis

Quantitative data were analysed in Stata (version 15.0). The prevalence of VI and blindness were not calculated for this study as the pilot study was not designed to provide a large enough sample size for meaningful estimates. The results of the RAHL study are reported separately. For time variables, medians and interquartile ranges (IQR) were obtained for skewed data, and means for normally distributed data. If the time of a questionnaire was greater than one hour, equal to zero, or a negative value for any of the examinations, these were excluded from the analysis. Feedback from field workers was summarised narratively.

### Ethics

The research followed the tenets of the Declaration of Helsinki. Ethical approval for the study was granted by the London School of Hygiene & Tropical Medicine (reference: 6207), the Public Health Foundation of India Institutional Ethics Committee (India) (reference: 84/2012), the Government of India Health Ministry Screening Committee (India) (reference: 36/ADR/2013-NCD-1), National Ethics Committee for Research in Human Health (Cameroon) (reference: 2013/03/084/CNERSH/SP), the Cameroon Baptist Convention Health Board Institutional Review Board (Cameroon) (reference: IRB2013-07), and the College of Medicine Research Ethics Committee (Malawi) (reference: P.03/18/2375).

Relevant health services available in each region were identified prior to the survey. People identified as having a need for ear and hearing, or eye care services were referred. Informed written/thumbprint consent was obtained from all study participants.

## Results

### Relationship between VI and HI in India and Cameroon

Prevalence of moderate or greater bilateral HI among people aged 50+ in India and Cameroon was 17.4% (95%CI 14.5, 20.7) and 14.8% (95%CI 11.5, 18.8), respectively. The prevalence of moderate or greater bilateral VI in people aged 50+ was in India 14.9% (95%CI 11.4, 19.3) and Cameroon 10.9% (95%CI 8.3, 14.3). The prevalence of combined VI and HI among people aged 50+ was 4.4% (95%CI 3.0, 6.4) in India and 4.8% (95%CI 3.0, 7.7) in Cameroon. Approximately one third of participants 50+ in India (29.3%) and Cameroon (36.2%) who had VI also had moderate or greater HI. Furthermore, approximately a quarter of participants in India (25.4%) and Cameroon (26.9%) who had HI, also had VI.

After adjusting for age and sex, people with moderate or worse VI had approximately double the odds of moderate or greater HI (and vice versa) in India (1.9; 95%CI 1.2, 3.0) and Cameroon (2.3; 95%CI 1.3, 4.4), respectively.

### Pilot study in Malawi

Across nine clusters, 256 people completed the RAHL protocol assessments of 270 eligible (response rate 94.8%). Of these, 188 participants completed the RAAB protocol (73.4%; missing data: n = 68). There were clear reasons that fewer people completed RAAB examinations compared to RAHL. For three clusters the response rate on first visit was relatively low, therefore repeat visits at the end of the survey were conducted. However, the RAAB component was not included in these repeat visits, resulting in missing vision data (missing data: n = 25; 9.7%). For three clusters, due to long travel times, two RAHL teams were required to complete one cluster. As this pilot study only included one RAAB team, only half of the participants could be included (missing data: n = 41; 16.0%). These issues are a result of having one RAAB team and only including the combined protocol in nine clusters (due to pilot study design). There were only two (0.8%) missing people in clusters where these issues did not occur. The OCO missed these participants as the combined survey team became spread out across the village and communicating the location of enrolled people was difficult.

#### Exam duration

The median total time per person for the RAHL component was 16.7 minutes (IQR = 5.2). The median duration of an E chart RAAB exam and questionnaire was 4.0 minutes (IQR = 5.0) per participant. The median time to complete consent (for both hearing and vision assessments) was 7 minutes (range 5–14 minutes). Including time taken for consent the RAAB-RAHL combined took a total median time of 27.5 minutes (IQR = 5.7) per participant. Table 1 shows a trend for increasing exam duration with increasing severity of both VI and HI. With a cluster size of 30 it was feasible to complete both protocols in one day.

**Table 1:** Average duration of examinations (assessment of VI and HI and causes) by degree of impairment (excluding consent).

	RAAB examinations (VI)			RAHL examinations (HI)		
Degree of HI or VI	n	median (mins)	Q1-Q3 (mins)	n	median (mins)	Q1-Q3 (mins)
Normal	101	3.0	2.0–5.0	586	15.6	13.9–17.9
Mild	14	6.0	3.0–16.0	302	17.7	15.8–20.5
Moderate	17	8.0	6.0–11.0	71	19.5	17.7–22.6
Severe	5	14.0	4.0–15.0	34	21.7	18.2–26.0
Blind/Profound	4	15.0	10.0–16.0	3	22.0	20.1–26.6
All	141	4	2–7	993		

n = 142 for examinations using E chart, 1 observation missing start time data.

#### Qualitative feedback

Fieldworkers reported that the combination of hearing and vision screening worked well even in a challenging setting, and did not add substantial time to the RAHL protocol. The OCO working alone noted that there were occasions where the RAAB component lagged behind the RAHL component; this was not unmanageable but would be alleviated by the addition of a second RAAB team member.

The survey teams felt that hearing and vision screening was positively received by study participants. People in the community were keen to have VI and HI screening, including those who were not part of the sample. Therefore, after the survey was completed screening camps in the district were arranged to meet the demand. Although not powered to estimate the prevalence of VI or eye disease, the survey highlighted a potential need for eye care services alongside hearing services in the rural areas of Ntcheu district. A full population-based survey is warranted to determine the needs and unmet needs for eye care services.

#### Budget comparison

Table 2 shows the costs of the four different scenarios and the cost savings that are possible through combining the two methodologies (e.g. costs of combining transport, training, and preparation). The cost of combining the RAAB and RAHL and completing the survey in all clusters with a sample size of 3,500 is 11% cheaper than conducting two stand-alone surveys. A more direct comparison on the relative costs is a combination of RAAB and RAHL but completing RAHL only in a third of clusters to suit the different sample sizes required is 28% cheaper. The third option, conducting a RAHL with the addition of VI assessment (i.e. not a RAAB) survey would be the cheapest option for a combined impairment screen due to the reduced sample size required.

**Table 2:** Cost of conducting combined RAAB and RAHL with different scenarios.

Scenario		RAAB and RAHL separately	RAAB-RAHL in all clusters	RAAB in all clusters, RAHL in ~1/3 clusters	RAHL with VI estimate only
Powered to detect		% moderate VI, blindness; % moderate HI			% moderate VI,; % moderate HI
Sample size*	RAHL	1100	3500	1100	1100
	RAAB	3500	3500	3500	1100
Cluster size**	RAHL	30	30	30	30
	RAAB	50	30	30	30
No. teams^	RAHL	2	4	2	2
	RAAB	4	4	4	2
No. field days	RAHL	18	30	18	18
	RAAB	18	30	30	18
No. team members^^	RAHL	4	4	4	4
	RAAB	2	2	2	2
Cost (GBP)		60,566	53,620	43,310	38,011
Cost reduction (%)		Comparator	11% cheaper	28% cheaper	37% cheaper

Assumptions

*Sample size calculation: RAHL based on 11.5% prevalence; 10% non-response; 20% margin of error; 95% confidence; design effect of 1.5; RAAB based on 3.3% prevalence; 10% non-response; 20% margin of error; 95% confidence; design effect of 2. The sample size is not fixed and will vary depending on the expected prevalence.

**Cluster size: Cluster size for RAAB is typically 50 and for RAHL 30. For combined surveys the cluster size is reduced to 30 based on the pilot study which found it is feasible to examine 30 people per day with a combined protocol.

**^Number of teams**: The number of teams used for the calculations is based on the sample size. The RAAB sample size is typically larger, and commonly 4 teams collect data over 4–5 weeks. The RAHL sample size is lower, and two teams can complete data collection over 4–5 weeks. When RAAB and RAHL are combined in all clusters (option 2), the number of RAHL teams is increased to match the number of RAAB teams. When RAHL is combined with a VI survey (option 4) the number of vision teams is reduced to match the number of RAHL teams based on the reduced sample size. However, the number of teams is not fixed and can vary depending on available resources.

**^^Number of team members:** RAAB team is typically two people (one person to test visual acuity; and one person to examine eyes). Number of team members for RAHL is four (one enumerator; two hearing testers; and one ear examiner.

## Discussion

### Key findings

This is the first pilot of a combined RAAB-RAHL survey protocol, and results inform the feasibility and possible cost-savings of combined rapid surveys of sensory impairment.

To assess the potential value of a combined impairment survey, we conducted a secondary data analysis of disability surveys conducted in India and Cameroon. This analysis showed that approximately one third of people with moderate or greater VI are also found to have moderate or greater HI in the 50+ age group. Furthermore, a quarter of people with HI also had VI. Compared to previous research in high-income countries the overlap between VI and HI in these two LMICs was lower. An Australian study found that 62.5% of people with VI had HI compared to a third in our study.[12] This may be due to differences in measurement techniques, survey protocol, and impairment definitions. Surveys in high income contexts have typically included early VI (<6/12) in their calculations, whereas we have used WHO definitions of moderate or greater impairment.[12] However, the overlap between the two impairments is still substantial and provides a rationale for a combined impairment population-based survey in order to maximise on minimal resources available for surveys of sensory impairments.

The pilot study in which RAHL was combined with RAAB in nine clusters, suggest that a combined approach is feasible and well accepted by participants and data collectors. Furthermore, undertaking a combined RAAB and RAHL is considerably cheaper than undertaking two separate surveys–by up to 29%. Examination duration for RAAB and RAHL combined was approximately 27 minutes per participant, and it was possible to examine 30 people for both VI and HI in one day. In the pilot study, no participants with VI were dilated. However, we would expect that on average 10% of RAAB participants may need dilation for examination of the posterior segment which could add up to 30 minutes examination time per person. We therefore estimate that adding RAAB to RAHL will add approximately one hour of fieldwork time per day compared to a RAHL only survey. The team may therefore need to make adaptations–such as RAHL team members could assist with uploading data to the server to minimise end of day delays.

### Implications

The idea of a combined vision and hearing surveys is not new. Several surveys combining the approaches have been conducted–mostly in high-income countries [31, 32], with a few also conducted in LMICs.[33, 34] Many more combine impairment screening in children only–such as in Key Informant Method studies in Bangladesh, Malawi, and other settings.[35, 36] However, a standardised, rapid approach for older adults in LMICs has not been developed by agencies such as the WHO. In the context of scarce resources for population-based surveys of HI and VI in LMICs, there is an opportunity to collect high quality data using a combined RAAB-RAHL.

The results of this study suggest that a combined approach would be feasible. However, further pilot testing is needed with a full RAAB team. There is an option in RAAB to include a module on diabetic retinopathy (RAAB DR). This methodology is more resource intensive than the standard RAAB due to the additional clinical examinations needed. To allow time for the additional examinations, a cluster size of 35 is used instead of 50. Currently, RAAB DR is not as commonly used as the standard RAAB however, in the context of increasing prevalence of non-communicable diseases in LMICs, it may become more common.[6] Thus, testing the feasibility and examining the cost implications of a RAAB DR and RAHL in combination is warranted.

Conducting surveys that measure more than one impairment is a better use of resources, improving efficiency and broadens the potential funding pool. Both RAHL and RAAB are standardised and tested approaches, intended to be used for planning purposes at a sub-national level. Furthermore, planning for eye care services may be by the same agencies, or closely connected groups within the Ministry of Health, in many countries. This is recognised in the recent World Report on Vision, which emphasises the need for eye health planning to be coordinated across sectors.[37]

### Limitations

Regarding the RAAB component of the survey, some deviations from the standard protocol were made. Firstly, a single OCO was used instead of a two-person team–the recommended approach in RAAB6. However, this was deemed appropriate due to RAHL cluster size of 30 compared to standard RAAB cluster size of 50. Budgetary constraints also prevented the use of an ophthalmologist in place of the OCO and therefore dilated eye exams (to assess posterior segment) were not carried out. This may have led to underestimates of the time taken to include RAAB. A further pilot including two RAAB team members and dilation of participants (as required) is warranted. Secondly, duration data was based on the opening and saving of the ODK forms and may not accurately reflect the duration of all exams. Some may have been opened prior to starting an exam to record participant details, others may have been counselled and given basic medication before forms were saved. We also made an estimation of the time taken for consent on a small number of field observations, which may have over or underestimated the time taken to complete the full examination.

Some caution is warranted in the interpretation of the cost estimates. We used cost data from a RAHL undertaken in one setting only and RAAB costs from 2013 estimates, inflated to today’s costs. In reality, the estimated costs will vary depending on the setting and time, number of teams, sample size and variation in local costs between settings. We aimed to demonstrate the potential relative cost savings from combining the approaches, not to present actual costs of conducting the survey.

Finally, the feasibility study is based on a small sample of people across nine clusters. Even within clusters the combined protocol was not completed on all participants for reasons outlined above. Further piloting in another setting may be warranted.

## Conclusion

VI and HI are common in older populations, and there is a substantial number of individuals with dual sensory impairment. Data from India and Cameroon analysed in this study suggest that this is also the case in LMICs. This pilot study of a combined RAAB-RAHL survey suggests the approach is feasible and lower cost than conducting two standalone impairment surveys. This combined survey may be a way to maximize limited resources to increase prevalence data for both vision and hearing impairment to be used for planning services.

## Supporting information

S1 File Rapid Assessment of Hearing Loss questionnaire—Malawi. (DOCX)

S2 File Rapid Assessment of Avoidable Blindness questionnaire. (PDF)

S3 File Population-based survey of disability questionnaire India (Mahabubnagar District). (XLSX)

S4 File Population-based survey of disability questionnaire Cameroon (Fundong Health District). (XLSX)
