# Supplementary material for: Rationale and feasibility of a combined rapid assessment of avoidable blindness and hearing loss protocol
Source: PLoS One. 2020 Feb 13;15(2):e0229008. doi: 10.1371/journal.pone.0229008 (PMC7018009; doi:10.1371/journal.pone.0229008)
Supplement: S1 File — (DOCX) [file pone.0229008.s001.docx]

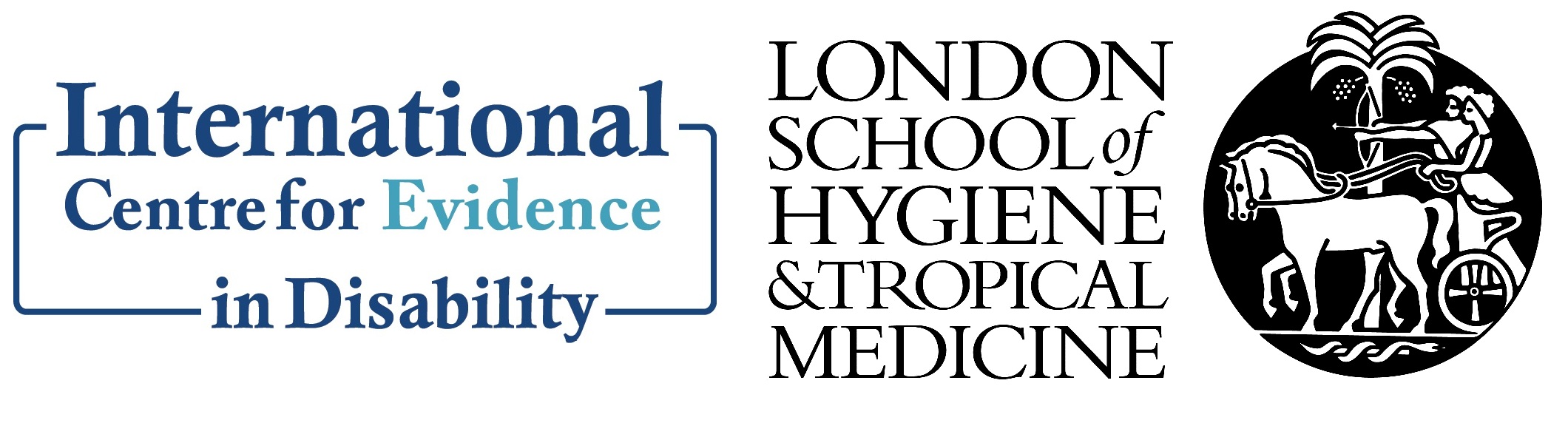


## **Rapid Assessment of Hearing Loss study: Questionnaire set**

**Please note: This questionnaire set is the paper-based version of what we will collect on mobile data collection platform (Open Data Kit), the skip patterns will be automated. Some questions will need to be adapted to the local setting.**

**Household roster**

This is completed for each household to keep track of the examination and referral status between team members

| **ID number** | **Name** | **Age** | **Sex** | **Is the participant available, not available, or did they refuse?** | **Questions complete** | **Self-report** | **Hearing test complete** | **Hearing test results (average threshold and degree L and R)** | **Otoscopy complete** | **Ear disease present (specify ear)?** | | | **Management provided in field (wax, medication, dry mop)** | | | **Referral made*** |
| --- | --- | --- | --- | --- | --- | --- | --- | --- | --- | --- | --- | --- | --- | --- | --- | --- |
|  |  |  |  |  |  |  |  |  |  |  | | |  | | |  |
|  |  |  |  |  |  |  |  |  |  |  | | |  | | |  |
|  |  |  |  |  |  |  |  |  |  |  | | |  | | |  |
|  |  |  |  |  |  |  |  |  |  |  | | |  | | |  |
|  |  |  |  |  |  |  |  |  |  |  | | |  | | |  |
|  |  |  |  |  |  |  |  |  |  |  | | |  | |  |  |
| ***Referral criteria** | |  |  |  |  |  |  |  |  |  | |  |  |  |  |  |
| Disabling hearing loss  Any ear disease in either ear that cannot be managed in the field  Mild hearing loss in both ears + self reported difficulties (a lot or greater)  Mild hearing loss in one ear, moderate or worse in the other ear | | | | | | | | | |  |  | | |  |  |  |
|  |  |  |  |  |  |  |  |  |  |  | | |  | |  |  |
|  |  |  |  |  |  |  |  |  |  |  | | |  | |  |  |
|  |  |  |  |  |  |  |  |  |  |  | | |  | |  |  |

|  | **SECTION A: GENERAL QUESTIONNAIRE** | | | | | | | | | | | | | | | |  |  |  |  |  |  |  |
| --- | --- | --- | --- | --- | --- | --- | --- | --- | --- | --- | --- | --- | --- | --- | --- | --- | --- | --- | --- | --- | --- | --- | --- |
| **Number** | **Question** | | | | | | | | | | | | | | **Response** | | **Skip pattern** |  |  |  |  |  |  |
| A1 | Date (DD/MM/YY)  ***Tsiku (DD/MM/YY)*** | | | | | | | | | | | | | |  | |  |  |  |  |  |  |  |
| A2 | Interviewer name  ***Dzina la yemwe akufunsa mafunso mukafukufukuyu*** |  | |  | | |  | |  | | |  | |  | ✍ | |  |  |  |  |  |  |  |
| A3 | Cluster name |  | |  | | |  | |  | | |  | |  | ✍ | |  |  |  |  |  |  |  |
|  | ***Dzina la gulu*** |  | |  | | |  | |  | | |  | |  |  | |  |  |  |  |  |  |  |
| ~~A4~~ | ~~Participant name~~  ***~~Dzina la yemweakutenga nawo mbali mukafukufukuyu.~~*** |  | |  | | |  | |  | | |  | |  | ~~✍~~ | |  |  |  |  |  |  |  |
| A5 | Participant initials  ***Dzina la yemwe akutenga nawo mbali mukafukufukuyu.*** |  | |  | | |  | |  | | |  | |  | ✍ | |  |  |  |  |  |  |  |
| A6 | Sex  ***Ndiwa mkazi kapena wamwamuna*** |  | |  | | |  | |  | | |  | |  | 1=Male  2=Female  ***1=Mamuna***  ***2=Mkazi*** | |  |  |  |  |  |  |  |
| A7 | Age in completed years *Enter this response carefully!*  ***Zaka zomwe wakwanitsa yemwe akutenga nawo mbali mukafukufukuyu***. |  | |  | | |  | |  | | |  | |  | ✍ | |  |  |  |  |  |  |  |
| A8 | Cluster number  ***Nambala*** ***ya gulu lawo*** |  | |  | | |  | |  | | |  | |  | ✍ | |  |  |  |  |  |  |  |
| A9 | House No.  ***Nambala ya nyumba*** |  | |  | | |  | |  | | |  | |  | ✍ | |  |  |  |  |  |  |  |
| A10 | Individual number  ***Nambala ya yemwe akutenga nawo mbali mukafukufukuyu.*** |  | |  | | |  | |  | | |  | |  | ✍ | |  |  |  |  |  |  |  |
| A11 | Participant ID *6 digit number (Cluster, House, Roster number)* |  | |  | | |  | |  | | |  | |  |  | |  |  |  |  |  |  |  |
|  | ***Nambala ya yemwe akutenga nawo mbali mukafukufukuyu*** |  | |  | | |  | |  | | |  | |  |  | |  |  |  |  |  |  |  |
| A12 | Exam status (up to this question to be completed for all participants regardless of whether they are home or not)  ***Kodi wayezedwa*** |  | |  | | |  | |  | | |  | |  | 1=Examined  2=Refused 🡪  3=Absent  4=Unable to communicate  ***1= Wayezedwa***  ***2= Wakana kuyezedwa***  ***3= Sanabwere***  ***4=*** ***Sangathe kulumikizani nanu*** | | Go to A12.1 |  |  |  |  |  |  |
| A12.1 | Reason for refusal  ***Chifukwa chomwe wakanira*** |  | |  | | |  | |  | | |  | |  | 1=Too busy  2=Not interested  3=Too sick  4=Other (specify)  ***1= Watanganidwa***  ***2= Sakusangalatsidwa***  ***3= Wadwalika***  ***4= Chifukwa china (monga)*** | |  |  |  |  |  |  |  |
| A13 | Who is completing the questionnaire?  ***Yemwe akuyankha mafunsowa ndi ndani?*** |  | |  | | |  | |  | | |  | |  | 1=Participant | |  |  |  |  |  |  |  |
|  |  |  | |  | | |  | |  | | |  | |  | 2=Proxy  ***1= Yemwe akutenga nawo mbali mukafukufukum***  ***2= Womuimirira*** | |  |  |  |  |  |  |  |
| A15 | Number of household members 50 years and older  ***Anthu omwe ali ndi zaka 50 kapena kuosera apo mnyumba muno ndi angati?*** |  | |  | | |  | |  | | |  | |  |  | |  |  |  |  |  |  |  |
| A14 | Number of household members 18-49 years  ***Anthu omwe ali ndi zaka 18 kulekeza 49 mnyumba muno ndi angati?*** |  | |  | | |  | |  | | |  | |  |  | |  |  |  |  |  |  |  |
| A16 | Number of household members 0-17 years-  ***Anthu omwe sanakwane chaka chimodzi komanso osaposera zaka 17 omwe amakhala myumba muno.*** |  | |  | | |  | |  | | |  | |  |  | |  |  |  |  |  |  |  |
|  | **LITERACY AND EDUCATION** |  | |  | | |  | |  | | |  | |  |  | |  |  |  |  |  |  |  |
| A17 | Can you/[name] read well, a little or not at all?  ***Kodi mumatha kuwerenga bwino bwino,pang'ono kapena simumatha kuwerenga.*** |  | |  | | |  | |  | | |  | |  | 1=Not at all  2=A little  3=Well  ***1=Sindimatha kuwerenga ngakhale pang'ono***  ***2=Ndimatha pang'ono***  ***3=Ndimatha kuwerenga bwino bwino*** | |  |  |  |  |  |  |  |
| A18 | What is the highest level of education you attended?  ***Sukulu munalekeza kalasi chani?*** |  | |  | | |  | |  | | |  | |  | 1=Never attended  2=Attended primary school | |  |  |  |  |  |  |  |
|  |  |  | |  | | |  | |  | | |  | |  | 3=Attended secondary school | |  |  |  |  |  |  |  |
|  |  |  | |  | | |  | |  | | |  | |  | 4=Attended tertiary education  ***1= Sindinapiteko ku sukulu***  ***2= Ndinapitapo ku pulaimale sukulu***  ***3= Ndinapitako ku sukulu ya sekondale***  ***4= Ndinapitako sukulu ya ukachenjede*** | |  |  |  |  |  |  |  |
|  | **EMPLOYMENT** |  | |  | | |  | |  | | |  | |  |  | |  |  |  |  |  |  |  |
| A19 | Other than domestic work in the household have you done any work in the last 4 weeks?  ***Kupatula ntchito ya mnyumba palinso ntchito ina yomwe mwakhala mukugwira kwa masabata 4 apitawo?*** |  | |  | | |  | |  | | |  | |  | 1=Yes | | Go to A20 |  |  |  |  |  |  |
|  |  |  | |  | | |  | |  | | |  | |  | 2=No  ***1=Eya***  ***2=Ayi*** | |  |  |  |  |  |  |  |
| A20 | Did you/did [name] do any of the following activities during the last 4 weeks?  ***Mwagwirako ntchito monga izi kwa masabata anayi omwe apitawo?*** |  | |  | | |  | |  | | |  | |  | 1=Farming/rearing animals/fishing | |  |  |  |  |  |  |  |
|  |  |  | |  | | |  | |  | | |  | |  | 2=Services/selling | |  |  |  |  |  |  |  |
|  |  |  | |  | | |  | |  | | |  | |  | 3=Factory work | |  |  |  |  |  |  |  |
|  |  |  | |  | | |  | |  | | |  | |  | 4=Houseworker at someone’s house  ***1= Ulimi, kuweta ziweto, usodzi***  ***2= Kukonza zinthu/kugulitsa malonda***  ***3= Ntchito yaku fakitale***  ***4= Kugwira ntchito ya mnyumba*** | |  |  |  |  |  |  |  |
|  | **HOUSEHOLD CHARACTERISTICS** | | | | | | | | | | | | | | | | |  |  |  |  |  |  |
|  | **Does your household have:**  ***Kodi nyumba mwanu muli zinthu monga izi:*** | | **Yes=1 (Eya=1)** | | | | | | | | | | | | | **No=2 (Ayi=2)** |  |  |  |  |  |  |  |
| A21 | Radio  ***Wailesi*** | | 1 | |  |  | |  | | |  | |  | | | 2 |  |  |  |  |  |  |  |
|  | Television  ***Wailesi yakanema*** | | 1 | |  |  | |  | | |  | |  | | | 2 |  |  |  |  |  |  |  |
|  | Bed with mattress  ***Bedi ndi matiresi***  Sofaset  ***Mpando wa sofa*** | | 1  1 | |  |  | |  | | |  | |  | | | 2  2 |  |  |  |  |  |  |  |
| A22 | Does any member of this household own a mobile phone?  ***Kodi alipo yemwe ali ndi foni mnyumba mwanu muno?*** | | 1 | |  |  | |  | | |  | |  | | | 2 |  |  |  |  |  |  |  |
| A23 | Does any member of this household have a bank account?  ***Kodi alipo yemwe ali ndi buku la ku banki nyumba mwanu muno?*** | | 1 | |  |  | |  | | |  | |  | | | 2 |  |  |  |  |  |  |  |
| A25 a) | Observe the main material of the floor  ***Yanganani pansi nyumba kuti muone zipangizo zomwe anamangira pansi pa nyumba yawoyo.*** | | | | | | | | | | | | | | | 1=earth/sand |  |  |  |  |  |  |  |
|  |  |  |  |  |  |  |  |  |  |  |  |  |  |  |  | 2=cement |  |  |  |  |  |  |  |
|  |  |  |  |  |  |  |  |  |  |  |  |  |  |  |  | 3=other floor material  ***1=Dothi/mchenga***  ***2=Simenti***  ***3=Zipangizo zina zozirira nyumba*** |  |  |  |  |  |  |  |
| A25 b) | Observe the main material of the roof  ***Yang'anani zipangizo zomwe anamangira denga la nyumba*** | | | | | | | | | | | | | | | 1=Grass  2=Iron sheet  3=Other roof material  ***1= Masamba amitengo***  ***2= Achitsulo***  ***3=Zipangizo zina zofolelera denga*** |  |  |  |  |  |  |  |
| A26 | What type of fuel does your household mainly use for cooking?  ***Kodi mumagwiritsira kwambiri nkhuni pophika?*** | | | | | | | | | | | | | | | 1=Gas  2=Coal  3=Electric |  |  |  |  |  |  |  |
|  |  |  |  |  |  |  |  |  |  |  |  |  |  |  |  | 4=Other type of fuel  ***1=Nkhuni***  ***2=Mtundu wina wa mafuta*** |  |  |  |  |  |  |  |
|  |  | | | | | | | | | | | | | | |  |  |  |  |  |  |  |  |
|  | **HEALTH** | | | | | | | | | | | | | | | | |  |  |  |  |  |  |
| A27 | In general, would you say your/[name’s] health is  ***Ponena mwachidule tinganene kuti umoyo wanu ndi wabwino?*** | | | | | | | | | | | | | | | 1=Excellent  2=Very good  3=Good  4=Moderate  5=Poor  ***1= Ndi wopambana zedi***  ***2= Ndiwabwino kwambiri***  ***3= Ndiwabwino***  ***4= Ndiwapakati ndipakati***  ***5= Siwabwino*** |  |  |  |  |  | 1=Excellent |  |
| **RISK FACTOR SCREEN** | | | | | | | | | | | | | | | | | |  |  |  |  |  |  |
| A28 | Have you ever been involved in work or non-work related activities where you were exposed to sounds or noise for 4 or more hours a day, several days per week?  *Loud noise means so loud that you must speak in a raised voice to be heard*  ***Munayamba mwagwira ntchito kapena munayamba mwapezeka pa malo poti panali phokoso kwanthawi yokwana maola anayi kapena kuposera apo patsiku kapena kwa masiku angapo musabata limodzi?#***  ***Phokoso lake lokupangitsani kuti mudzilankhula mokweza kuti mumvane ndi anthu?*** | | | | | | | | | | | | | | | 1=Yes 🡪 | Go to A29 |  |  |  |  |  |  |
|  |  |  |  |  |  |  |  |  |  |  |  |  |  |  |  | 2=No 🡪  ***1=Eya***  ***2=Ayi*** | Go to A30 |  |  |  |  |  |  |
| A29 | For how long have you been exposed at work to loud sounds for more than 4 hours a day, several days a week?  ***Kodi munagwira ntchito kwanthawi yayitali bwanji pamalo a phokoso la makina lomwe linali lopitilira maola anayi pa tsiku?*** | | | | | | | | | | | | | | | 1=Less than 1 year  2=1 year or more  ***1= Nthawi yosachepera chaka chimodzi***  ***2= Chaka chimodzi kapena kupitilira apo*** |  |  |  |  |  |  |  |
| A30 | Do/did you use noise protection when you are exposed to very loud noise?  ***Munkhavala zipangizo zodzitetezera pa nthawi yomwe mumapezeka pa malo aphokoso lambiri?*** | | | | | | | | | | | | | | | 1=Yes  2=No  ***1=Eya***  ***2=Ayi*** |  |  |  |  |  |  |  |
| A31 | Have you ever been involved in work or non-work related activities where you were exposed to chemicals (examples of where chemical exposure include in: paint manufacture, adhesive manufacture, paint stripping, plastics manufacture, electroplating, and laboratory chemicals)?  ***Munayamba mwagwira ntchito pa malo pomwe pamapezeka mankhwala monga omwe amagwiritsidwa ntchito ku kampani yopanga simenti, zipangizo zomatila, yokonza zinthu za pulasitiki,zipangizo zomwe amagwiritsira ntchito ku labotale kapena zipangizo za magetsi?*** | | | | | | | | | | | | | | | 1=Yes  2=No  ***1=Eya***  ***2=Ayi*** |  |  |  |  |  |  |  |
| A32 | Do you have a history of medication use for cancer (chemotherapy)?  ***Munayamba mwalandira mankhwala a kansa?*** | | | | | | | | |  | | | | | | 1=Yes 🡪 | Go to A32.1 |  |  |  |  |  |  |
|  |  |  |  |  |  |  |  |  |  |  | | | | | | 2=No 🡪  ***1=Eya 🡪***  ***2=Ayi 🡪*** | Go to A33 |  |  |  |  |  |  |
| A32.1 | Did you notice a change in your hearing when you started to use this medication?  ***Mwaonako kusintha kwina kulikonse kwa mamvedwe chiyambireni kumwa mankhwala amenewa?*** | | | | | | | | |  | | | | | | 1=Yes |  |  |  |  |  |  |  |
|  |  |  |  |  |  |  |  |  |  |  | | | | | | 2=No  ***1=Eya***  ***2=Ayi*** |  |  |  |  |  |  |  |
| A33 | Have you ever been told by a doctor/nurse that you have diabetes, sugar in your urine or high blood sugar?  ***Munayamba mwadwala matenda a shuga?*** | | | | | | | | |  | | | | | | 1=Yes |  |  |  |  |  |  |  |
|  |  |  |  |  |  |  |  |  |  |  | | | | | | 2=No  ***1=Eya***  ***2=Ayi*** |  |  |  |  |  |  |  |
| A34 | Have you been told by your doctor/nurse that you have high blood pressure?  ***Munayamba mwauzidwa ndi dokotala wanu zoti muli ndi nthenda ya BIPI?*** | | | | | | | | |  | | | | | | 1=Yes |  |  |  |  |  |  |  |
|  |  |  |  |  |  |  |  |  |  |  | | | | | | 2=No  ***1=Eya***  ***2=Ayi*** |  |  |  |  |  |  |  |
| A35 | Do you have a history of head injury?  ***Munayamba mwavulala m'mutu?*** | | | | | | | | |  | | | | | | 1=Yes 🡪 | Go to A35.1 |  |  |  |  |  |  |
|  |  |  |  |  |  |  |  |  |  |  | | | | | | 2=No 🡪  ***1=Eya***  ***2=Ayi*** | Go to A36 |  |  |  |  |  |  |
| A35.1 | Did you notice a change in your hearing after your injury?  ***Munaona kusintha kwamamvedwe potsatira kuvulala kwam'mutuko?*** | | | | | | | | |  | | | | | | 1=Yes |  |  |  |  |  |  |  |
|  |  |  |  |  |  |  |  |  |  |  | | | | | | 2=No  ***1=Eya***  ***2=Ayi*** |  |  |  |  |  |  |  |
| A36 | Does anyone in your immediate family have a hearing loss since childhood?  ***M'banja mwanu alipo yemwe anapezeka ndi vuto lakusamvetsetsa kuyambira ali mwana?*** | | | | | | | | |  | | | | | | 1=Yes 🡪 | Go to A36.1 |  |  |  |  |  |  |
|  |  |  |  |  |  |  |  |  |  |  | | | | | | 2=No 🡪  ***1=Eya***  ***2=Ayi*** | Go to A37 |  |  |  |  |  |  |
| A36.1 | Relative with hearing loss  ***Muli ndi m'bale yemwe samamvetsetsa?*** | | | | | | | | |  | | | | | | 1=Parent |  |  |  |  |  |  |  |
|  |  |  |  |  |  |  |  |  |  |  | | | | | | 2=Sibling |  |  |  |  |  |  |  |
|  |  |  |  |  |  |  |  |  |  |  | | | | | | 3=Child  ***1=Kholo***  ***2=Mchemwali kapena mchimwene wake***  ***3=Mwana*** |  |  |  |  |  |  |  |
| A37 | In the past 12 months, have you been bothered by ringing or buzzing noises in your ears that lasts for 5 minutes or more?  ***Kwa miyezi khumi ndi iwiri yapitazo mmakutu mwanu mwakhala mukumveka kulira kwa mphindi zokwana zisanu kapena kuposera apo?*** | | | | | | | | |  | | | | | | 1=Yes 🡪 | Go to A37.1 |  |  |  |  |  |  |
|  |  |  |  |  |  |  |  |  |  |  | | | | | | 2=No 🡪  ***1=Eya***  ***2=Ayi*** | Go to A38 |  |  |  |  |  |  |
| A37.1 | In the 12 months, how often have you experienced this ringing?  ***Kwa mwezi khumi ndiiwiri yapitayo mwakhala mukumva kulira mkhutu mwanu mowirikiza bwanji?*** | | | | | | | | |  | | | | | | 1= Almost always |  |  |  |  |  |  |  |
|  |  |  |  |  |  |  |  |  |  |  | | | | | | 2= Once a day |  |  |  |  |  |  |  |
|  |  |  |  |  |  |  |  |  |  |  | | | | | | 3= Once a week |  |  |  |  |  |  |  |
|  |  |  |  |  |  |  |  |  |  |  | | | | | | 4= Once a month |  |  |  |  |  |  |  |
|  |  |  |  |  |  |  |  |  |  |  | | | | | | 5= Less often than once/month  ***1=Pafupi fupi nthawi zonse.***  ***2=Kamodzi pa tsiku***  ***3=Kamodzi pa sabata***  ***4=Kamodzi pa mwezi***  ***5= Nthawi yosachepera kamodzi pa mwezi.*** |  |  |  |  |  |  |  |
|  | **Do you have a history of any of the following illnesses (adapted based on country setting):** | | | | | | | | | **Yes** | | | | | | **No** |  |  |  |  |  |  |  |
| A38 | Tuberculosis  ***TB*** | | | | | | | | | 1 🡪 | | | | | | 2 | Answer A41 |  |  |  |  |  |  |
|  | Meningitis  ***Matenda oumitsa khosi*** | | | | | | | | | 1 | | | | | | 2 |  |  |  |  |  |  |  |
|  | Pneumonia  ***Chibayo*** | | | | | | | | | 1 | | | | | | 2 |  |  |  |  |  |  |  |
|  | Herpes Zoster (Shingles)  ***Mashingozi*** | | | | | | | | | 1 | | | | | | 2 |  |  |  |  |  |  |  |
|  | Chicken pox  ***Katsabola*** | | | | | | | | | 1 | | | | | | 2 |  |  |  |  |  |  |  |
|  | Syphilis  ***Chindoko*** | | | | | | | | | 1 | | | | | | 2 |  |  |  |  |  |  |  |
|  | Mumps  ***Matsagwidi*** | | | | | | | | | 1 | | | | | | 2 |  |  |  |  |  |  |  |
|  | Measles  ***Chikuku*** | | | | | | | | | 1 | | | | | | 2 |  |  |  |  |  |  |  |
|  | Malaria  ***Malungo*** | | | | | | | | | 1🡪 | | | | | | 2 | Answer A39 |  |  |  |  |  |  |
|  |  | | | | | | | | | 1 | | | | | | 2 |  |  |  |  |  |  |  |
|  | HIV/AIDS  ***AIDS*** | | | | | | | | | 1 🡪 | | | | | | 2 | Answer A40 |  |  |  |  |  |  |
| A39 | Did you receive medication for the malaria?  ***Munayamba mwalandira mankhwala a malungo?*** | | | | | | | | |  | | | | | | 1=Yes  2=No  ***1=Eya***  ***2=Ayi*** |  |  |  |  |  |  |  |
| A40 | Do you take medication for the HIV/AIDS?  ***Mumamwa ma ARV?*** | | | | | | | | |  | | | | | | 1=Yes  2=No  ***1=Eya***  ***2=Ayi*** |  |  |  |  |  |  |  |
| A41 | Do you take medication for the TB?  ***Mumamwa mankhwala a TB?*** | | | | | | | | |  | | | | | | 1=Yes  2=No  ***1=Eya***  ***2=Ayi*** |  |  |  |  |  |  |  |
| A42 | Did you notice a change in your hearing after this illness?  ***Kodi mwazindikira kuti kusamvetsetsa kwanu kwaonjezereka pambuyo poti munadwala?*** | | | | | | | | |  | | | | | | 1=Yes  2=No  ***1=Eya***  ***2=Ayi*** | If answered yes to any 59-74 |  |  |  |  |  |  |

**Interviewer: Take note if this person self-reports hearing loss, or other difficulties (seeing, hearing, self-care, communication, walking, remembering or concentrating)**

|  | | **Section B: HEARING SCREENING (note that this data is input directly in to the form from the hearTest app)** | | | | | | | | | | | |  |  |
| --- | --- | --- | --- | --- | --- | --- | --- | --- | --- | --- | --- | --- | --- | --- | --- |
| **Audiometry** | | | | | | | | | | | | | | | |
| B1 | Date (DD/MM/YY)  ***Tsiku (DD/MM/YY)*** | | | | | | | | | | |  | | | |
| B2 | Interviewer name  ***Dzina la yemwe akufunsa mafunso mukafukufukuyu*** | |  | |  |  | |  |  | |  | ✍ | | | |
| B3 | Cluster name | |  | |  |  | |  |  | |  | ✍ | | | |
|  | ***Dzina la gulu; Dzina la mudzi*** | |  | |  |  | |  |  | |  |  | | | |
| B4 | Participant name  ***Dzina la yemwe akutenga nawo mbali mukafukufukuyu.*** | |  | |  |  | |  |  | |  | ✍ | | | |
| B5 | Participant initials  ***Dzina lake mwachidule*** | |  | |  |  | |  |  | |  | ✍ | | | |
| B6 | Sex  ***Ndiwa mkazi kapena wamwamuna*** | |  | |  |  | |  |  | |  | 1=Male  2=Female  ***1=Mamuna***  ***2=Mkazi*** | | | |
| B7 | Age in completed years *Enter this response carefully!*  ***Zaka zomwe wakwanitsa yemwe akutenga nawo mbali mukafukufukuyu***. | |  | |  |  | |  |  | |  | ✍ | | | |
| B8 | Cluster number  ***Nambala*** ***ya gulu lawo*** | |  | |  |  | |  |  | |  | ✍ | | | |
| B9 | House No.  ***Nambala ya nyumba*** | |  | |  |  | |  |  | |  | ✍ | | | |
| B10 | Individual number  ***Nambala ya wotenga mbali mukafukufukuyu.*** | |  | |  |  | |  |  | |  | ✍ | | | |
| B11 | Participant ID *6 digit number (Cluster, House, Roster number)* | |  | |  |  | |  |  | |  |  | | | |
|  | ***Nambala ya yemwe akutenga nawo mbali mukafukufukuyu*** | |  | |  |  | |  |  | |  |  | | | |
| B12 | **Exam status** | |  | |  |  | |  |  | |  | 1=Complete  2=Unable to test | | | |
| B13 | **Start hearing test button** | |  | |  |  | |  |  | |  | (link to hearTest app) | | | |
|  | *The below details will be automatically input from hearTest to ODK* | | | | | | | | | | | | | | |
|  | |  | | **Left ear** | | | | | | **Right ear** | | | |  |  |
|  | | Frequency (Hertz) | | Threshold (decibels, dB) | | | | | | | | | |  |  |
|  | | 500 | |  | | | | | |  | | | |  |  |
|  | | 1000 | |  | | | | | |  | | | |  |  |
|  | | 2000 | |  | | | | | |  | | | |  |  |
|  | | 4000 | |  | | | | | |  | | | |  |  |
|  | |  | |  | | |  | | |  | | |  |  |  |
|  | | Pure tone average | | EAVGL | | |  | | | EAVGR | | |  |  |  |
|  | | *Average of thresholds at 500, 1000, 2000, 4000 Hz* | | | | | | | | | | | | | |
|  | | Noise concerns (provided by app) | | | | | | | | | | | | | |
|  | | Test re-test concerns (provided by app) | | | | | | | | | | | | | |

**Take note of the results on the roster**

**Next step: ear examination**

|  | **Section C: EAR EXAMINATION** | | | | | | | |  |
| --- | --- | --- | --- | --- | --- | --- | --- | --- | --- |
| C1 | Date (DD/MM/YY)  ***Tsiku (DD/MM/YY)*** | | | | | |  | |  |
| C2 | Interviewer name  ***Dzina la yemwe akufunsa mafunso mukafukufukuyu*** |  |  |  |  |  | ✍ |  |  |
| C3 | Cluster name |  |  |  |  |  | ✍ |  |  |
|  | ***Dzina la gulu; Dzina la mudzi*** |  |  |  |  |  |  |  |  |
| C4 | Participant name  ***dzina yemwe akutenga nawo mbali mukafukufukuyu.*** |  |  |  |  |  | ✍ |  |  |
| C5 | Participant initials  ***dzina la yemwe akutenga mbali mwachidule.*** |  |  |  |  |  | ✍ |  |  |
| C6 | Sex  ***Ndiwa mkazi kapena wamwamuna*** |  |  |  |  |  | 1=Male  2=Female  ***1=Mamuna***  ***2=Mkazi*** | |  |
| C7 | Age in completed years *Enter this response carefully!*  ***Zaka zomwe wakwanitsa yemwe akutenga nawo mbali mukafukufukuyu***. |  |  |  |  |  | ✍ |  |  |
| C8 | Cluster number  ***Nambala*** ***ya gulu lawo*** |  |  |  |  |  | ✍ |  |  |
| C9 | House No.  ***Nambala ya nyumba*** |  |  |  |  |  | ✍ |  |  |
| C10 | Individual number  ***Namabala ya yemwe akutenga nawo mbali.*** |  |  |  |  |  | ✍ |  |  |
| C11 | Participant ID *6 digit number (Cluster, House, Roster number)*  ***Nambala ya yemwe akutenga nawo mbali mukafukufukuyu*** |  |  |  |  |  |  |  |  |
|  | **Hearing health history** | | | | | | | |  |
| C12 a) | Ask person who completed hearing test or check the roster: does this participant have a hearing loss in the right?  Pure tone average of >25dB HL  ***Mufunseni yemwe amayeza zamamvedwe ngati munthuyu mamvedwe ake ali otsika kukhutu lake lakumanja. Zotsatira zopitilira 25 dBhL*** | 1=Yes  2=No  ***1=Eya***  ***2=Ayi*** | | | | |  | |  |
| C12 b) | Ask person who completed hearing test or check the roster: does this participant have a hearing loss in the left?  Pure tone average of >25dB HL  ***Mufunseni yemwe amayeza zamamvedwe ngati munthuyu mamvedwe ake ali otsika kukhutu lake lakumanzere. Zotsatira zopitilira 25 dBHL*** | 1=Yes  2=No  ***1=Eya***  ***2=Ayi*** | | | | |  | |  |
| C13 | Do you own a hearing aid?  ***Kodi muli ndi makina omvera?*** | 1=Yes 🡪  2=No 🡪  ***1=Eya 🡪***  ***2=Ayi 🡪*** | | | | | Go to C14  Go to C18 | |  |
| C14 | Type of hearing aid  ***Mtundu wa makina omvera*** | 1=Hearing aid worn in pocket  2=Hearing aid worn behind the ear  3=Hearing aid worn in the ear  4=Unsure  ***1= Makina omvera oika mthumba***  ***2= Makina omvera ovala kumbuyo kwa khutu***  ***3= Makina omvera ovala mkati mwa khutu***  ***4= Sindikudziwa*** | | | | |  | |  |
| C15 | In the past year, how often have you worn your hearing aid?  ***Chaka chapitachi mwakhala mukuvala makina omvera kwa nthawi yayitali bwanji?*** | 1=Never/sindinamvalepo  2=Once a week/kamodzi pa sabata.  3=2-6 times a week/**kawiri kapena ka 6 pa sabata.**  **4=Every day/tsiku ndi tsiku** | | | | |  | |  |
| C16 | What is the main reason that you do not always wear your hearing aids?  ***Chifukwa chani simumavala makina omvera nthawi zonse?*** | 1) They are uncomfortable  2) They don’t help my hearing  3) They are broken  4) They were lost or stolen  5) I wear them when I go out  6) I don’t need to communicate all of the time  7) I feel embarrassed when wearing them  ***1= amandisowetsa mtendere.***  ***2= samathandiza kuti ndidzimva bwino***  ***3= anaonongeka***  ***4= anataika kapena kubedwa***  ***5= ndimavala ndikakhala pa ulendo.***  ***6= sindimafuna kulumikizana nthawi zonse.***  ***7= ndimachita manyazi ndikavala*** | | | | |  | |  |
| C17 | Think about the situation where you most wanted to hear better before you got your hearing aids.  ***Musanapeze makina omvera kumbukirani nthawi yomwe mumalakalaka kuti mumve bwino.***  Over the past two weeks, how much has your hearing aids helped in that specific situation? ***Kwa masabata awiri apitawo, makina anu akuthandizani motani munyengo yoteroyo.*** | 1) Helped not at all  2) Helped slightly  3) Helped moderately  4) Helped quite a lot  5) Helped very much  ***1= Sanandithandize.***  ***2= Anathandiza pang’ono.***  ***3= Anathandiza pakati ndi pakati.***  ***4= Amathandiza kwambiri***  ***5= Amathandiza kwambiri zedi.*** | | | | |  | |  |
| C18 a) | Do you have difficulty hearing?  ***Kodi mumavutika kuti mumve?*** | 1=No difficulty  2=Yes, some difficulty  3=Yes, a lot of difficulty  4=Cannot do at all  ***1=Ayi sindimavutika***  ***2=Eya ndimavutika pang'ono***  ***3=Eya ndimavutika kwambiri***  ***4=Sindimamveratu*** | | | | |  | |  |
| C18 b) | Do you have difficulty hearing, even if wearing your hearing aids?  ***Kodi mumavutika kumva ngakhale mutavala makina omvera?*** | 1=No difficulty  2=Yes, some difficulty  3=Yes, a lot of difficulty  4=Cannot do at all  ***1=Ayi sindimavutika***  ***2=Eya ndimavutika pang'ono***  ***3=Eya ndimavutika kwambiri***  ***4=Sindimamveratu*** | | | | |  | |  |
| C19 | How long have you experienced difficulties with your hearing?  ***Papita nthawi yayitali bwanji chiyambireni mukuvutika ndi vuto la kusamvetsetsa?*** | 1=Since birth  2=Since childhood  3=Since adulthood  4=Not known  5=Other (specify)  ***1= Chibadwireni***  ***2= Kuyambira ndili wang'ono***  ***3= Ndili wamkulu kale***  ***4=Sindikudziwa.***  ***5= Other (specify)*** | | | | |  |  |  |
| C20 | *For those who have hearing loss since adulthood*  Can you estimate how many years ago you started having difficulties?  ***Mongo yerekeza mungakumbukire kuti patha zaka zingati chiyambireni vuto lanu lakusamvetsetsa?*** | ✍ | | | | |  | |  |
| C21 | Was your hearing loss gradual or sudden?  ***Kodi vuto lakusamvetsetsali linangoyamba mwadzidzidzi kapena linayamba pang'ono pang'ono?*** | 1=Gradual  2=Sudden  ***1= Pang'ono pang'ono***  ***2= Mwadzidzidzi*** | | | | |  | |  |
| C22 | Did anything happen around the time your hearing loss started?  ***Pali china chake chomwe chinachitika panthawi yomwe mamvedwe anu munazindikira kuti akutsika?*** | 1=Yes  2=No  ***1=Eya***  ***2=Ayi*** | | | | |  | |  |
| C22.1 | Please specify what happened  ***Chonde fotokozani momwe zinakhalira.*** | ✍ | | | | |  | |  |
| C23 | Have you had surgery on your ears in the past?  ***Munayamba mwapangidwa opareshoni yamakutu*** | 1=Yes 🡪  2=No 🡪  ***1=Eya🡪***  ***2=Ayi🡪*** | | | | | Go to C24  Go to C25 |  |  |
| C24 | When was the surgery?  ***Opareshoni munapangidwa liti?*** | 1=3 months ago or less  2=More than 3 months ago  ***1= Mwezi itatu yapitayo kapena kuchepera***  ***2=Mwezi yoposa itatu*** | | | | |  | |  |
| C25 | Do you have a history of discharging ears?  ***Munayamba mwatuluka mafinya mkhutu?*** | 1=Yes  2=No  ***1=Eya***  ***2=Ayi*** | | | | |  |  |  |
| **EXAMINATION** | **For the LEFT and RIGHT ears** | | | | | | | |  |
| C26 | Does the participant have any ear pain? | 1=Yes in pinna  2=Yes in ear canal  3=Yes behind my ear  4=No | | | | |  | |  |
| C27 | **Pinna** | 1=Normal | | | | |  | |  |
|  |  | 2=Abnormal | | | | |  | |  |
|  | **Canal** | | | | | | | |  |
| C28 | Inflammation | 1=Yes | | | | |  | |  |
|  |  | 2=No | | | | |  | |  |
| C29 | Impacted wax | 1=Yes | | | | |  | |  |
|  |  | 2=No | | | | |  | |  |
| C30 | Foreign body | 1=Yes | | | | |  | |  |
|  |  | 2=No | | | | |  | |  |
| C31 | Discharge | 1=Yes🡪 | | | | | Go to C31.1 | |  |
|  |  | 2=No🡪 | | | | | Go to C32 | |  |
| C31.1 | Ask participant: how long have you had discharge? | 1=Less than 2 weeks | | | | |  | |  |
|  |  | 2=2 weeks or more | | | | |  | |  |
|  | **Tympanic membrane** | | | | | | | |  |
| C32 | Can the TM be seen? | 1=Yes 🡪 | | | | | Go to C33 | |  |
|  |  | 2=No 🡪 | | | | | Go to C39 | |  |
| C33 | Perforation | 1=Yes | | | | |  | |  |
|  |  | 2=No | | | | |  | |  |
| C34 | Shape | 1=Normal | | | | |  | |  |
|  |  | 2=Bulging | | | | |  | |  |
|  |  | 3=Retracted | | | | |  | |  |
| C35 | Colour | 1=Pearly white  2=Red  3=Pearly white | | | | |  | |  |
| C36 | Light reflex visible | 1=Yes  2=No | | | | |  | |  |
| C37 | **Middle ear** | 1=Normal | | | | |  | |  |
|  |  | 2=Fluid | | | | |  | |  |
|  |  | 3=Not seen | | | | |  | |  |
| C38 | Summary of ear examination | 1=Normal | | | | |  | |  |
|  |  | 2=Abnormal | | | | |  | |  |
| C39 | Is this abnormality likely to be the cause of hearing loss in this ear? | 1=Yes 2=No | | | | |  | |  |
| C40 | What is the diagnosis in this year? (based on the ear examination) | 1=AOM  2=OME  3=CSOM  4=Wax  5=FB  6=OE  7=DP  8=Normal  9=Other (specify) | | | | |  | |  |
| C41 | What is the likely cause of hearing loss in this ear? (if hearing loss present) | 1=Acute otitis media  2=Otitis media with effusion  3=Chronic suppurative otitis media  4=Impacted wax (fully occluding)  5=Foreign body  6=Otitis externa  7=Dry perforation  8=Infectious disease (e.g. meningitis, 9=HIV, malaria)  10=Noise exposure  11=Ototoxic medication  12=Congenital  13=Non-infectious disease (e.g. diabetes, cardiovascular disease)  14=Unknown | | | | |  | |  |
| C42 | What management did you provide for this participant? | 1=Treatment in the field, no referral 🡪  2=Treatment in the field and referral 🡪  3=Referral 🡪  4=No action🡪 | | | | | Go to C43  Go to C43  Go to C44  Go to C45 | |  |
| C43 | What treatment did you provide in the field | 1=Dry mop  2=Oral antibiotics  3=Topical antibiotics  4=Topical antifungal  5=Analgesics (e.g. paracetamol)  6=Wax removal  7=Foreign body removal | | | | |  | |  |
| C44 | What was the referral for? | 1=Diagnostic hearing evaluation and possible hearing aid fitting  2=Surgical assessment  3=Wax removal following wax drops  4=Other (specify) | | | | |  | |  |
| C45 | Ask participant: did your hearing improve following wax or foreign body removal? | 1) Yes  2) No  3) Not applicable | | | | |  | |  |
|  | **Additional questions** | | | | | | | |  |
| C45 | Does the participant have any other physical features on head and neck associated with hearing loss (e.g. skin tags, eye colour)? | 1=Yes 2=No | | | | |  | |  |
| C45.1 | Specify |  | | | | |  | |  |
|  | **Previous care seeking (questions for the participant)** | | | | | | | |  |
| C47 | Have you ever sought care for your ear or hearing difficulties?  ***Munayambapo mwafunafuna thandizo chifukwa cha vuto lomwe mulinalo lakusamvetsetsalo?*** | 1=Yes 🡪  2=No 🡪  ***1=Eya🡪***  ***2=Ayi🡪*** | | | | | Go to C48  Go to C50 | |  |
| C48 | If so where did you seek care?  ***Munakafuna kuti thandizolo?*** | 1=Health Centre  2=Public Hospital  3=Private clinic  4=Traditional provider  5=Pharmacist  6=Other (specify)  ***1=Chipatala chaching'ono***  ***2=Chipatala cha aliyense***  ***3=Chipatala cholipira***  ***4=Sing'anga***  ***5=Kwa dokolala yemwe anaphunzira zokhudza mankhwala***  ***5=Thandizo lina*** | | | | |  | |  |
| C49 | What treatment did you receive?  ***Munalandira thandizo lanji?*** | 1=Medication  2=Surgery  3=Assistive device  4=No treatment  5=Other (specify)  ***1=Mankhwala***  ***2=Opareshoni***  ***3=Machini omvera***  ***4=Sindinalandire thandizo***  ***5=Thandizo lina*** | | | | |  | |  |
| C50 | What was the reason that you have not sought care?  ***Chifukwa chani simunafunefune thandizo?*** | 1=Need not felt  2=Fear  3=Cannot afford treatment  4=Treatment denied by provider  5=Unaware that treatment is possible  6=No access to treatment  7=Other (specify)  1***=Sikunali kofunika***  ***2=Ndimaopa***  ***3=Sindinakwanitse kulipira kuti ndilandire thandizo.***  ***4=Anakana kundithandiza.***  ***5=Sindimadziwa kuti thandizo lilipo.***  ***6=Sindinakwanitse kufikira thandizo loyenera.***  ***7=Chifukwa china*** | | | | |  | |  |

**Interviewer: Take note if this person has a problem in either ear. Referrals for participants – provide counselling on referral and information sheet for those in need**

**Thank participant for their time. END QUESTIONNAIRE SET**
