# Supplementary material for: Rationale and feasibility of a combined rapid assessment of avoidable blindness and hearing loss protocol
Source: PLoS One. 2020 Feb 13;15(2):e0229008. doi: 10.1371/journal.pone.0229008 (PMC7018009; doi:10.1371/journal.pone.0229008)
Supplement: S2 File — (PDF) [file pone.0229008.s002.pdf]

RAPID ASSESSMENT FOR AVOIDABLE BLINDNESS

A. GENERAL INFORMATION

Year - month: ☐☐☐☐ - ☐☐

Survey area: ____________ ☐☐ **Cluster:** ☐☐☐ **Individual no.:** ☐☐

Name: ____________ **Sex:** Male: O (1) Female: O (2) **Age (years):** ☐☐

Optional 1: ☐☐
Optional 2: ☐☐

Examination status:

Examined:	O (1)	(go to B)	Refused:	O (3)	(go to E)
Not available:	O (2)	(go to E)	Not able to communicate:	O (4)	(go to E)

Always ask: "Did you ever have any problems with your eyes?" Yes: O (1) No: O (2)

If not available - details (availability / tel number / address)

B. VISION

Uses distance glasses: No: O (1) Yes: O (2)
Uses reading glasses: No: O (1) Yes: O (2)

**Presenting vision**	**Right eye**	**Left eye**
Can see 0,3	O (1)	O (1)
Cannot see 0,3 but can see 0,5	O (2)	O (2)
Cannot see 0,5 but can see 1,0	O (3)	O (3)
Cannot see 1,0 but can see 1,3	O (4)	O (4)
Cannot see 1,3 but can see 1,8	O (5)	O (5)
Light perception (PL+: 3)	O (6)	O (6)
No light perception (PL-: 4)	O (7)	O (7)

**Pinhole vision**	**Right eye**	**Left eye**
Can see 0,3	O (1)	O (1)
Cannot see 0,3 but can see 0,5	O (2)	O (2)
Cannot see 0,5 but can see 1,0	O (3)	O (3)
Cannot see 1,0 but can see 1,3	O (4)	O (4)
Cannot see 1,3 but can see 1,8	O (5)	O (5)
Light perception (PL+: 3)	O (6)	O (6)
No light perception (PL-: 4)	O (7)	O (7)

C. LENS EXAMINATION

	**Right eye**	**Left eye**
Normal lens / minimal lens opacity:	O (1)	O (1)
Obvious lens opacity:	O (2)	O (2)
Lens absent (aphakia):	O (3)	O (3)
Pseudophakia without PCO:	O (4)	O (4)
Pseudophakia with PCO:	O (5)	O (5)
No view of lens:	O (6)	O (6)

D. MAIN CAUSE OF PRESENTING VA>0.3

(Mark only one cause for each eye)

	**Right eye**	**Left eye**	**Principal cause in person**
Refractive error:	O (1)	O (1)	O (1)
Aphakia, uncorrected:	O (2)	O (2)	O (2)
Cataract, untreated:	O (3)	O (3)	O (3) (F)
Cataract surg. complications:	O (4)	O (4)	O (4)
Trachoma corneal opacity:	O (5)	O (5)	O (5)
Other corneal opacity:	O (6)	O (6)	O (6)
Phthisis:	O (7)	O (7)	O (7)
Onchocerciasis:	O (8)	O (8)	O (8)
Glaucoma:	O (9)	O (9)	O (9)
Diabetic retinopathy:	O (10)	O (10)	O (10)
ARMD:	O (11)	O (11)	O (11)
Other posterior segment:	O (12)	O (12)	O (12)
All globe/CNS abnormalities:	O (13)	O (13)	O (13)
Not examined: can see 0.3	O (14)	O (14)	O (14)

E. HISTORY, IF NOT EXAMINED

(From relative or neighbour)

**Believed**	**Right eye**	**Left eye**
Not blind	O (1)	O (1)
Blind due to cataract	O (2)	O (2)
Blind due to other causes	O (3)	O (3)
Operated for cataract	O (4)	O (4)

F. WHY CATARACT SURGERY WAS NOT DONE

(Mark up to 2 responses, if VA>0.5, not improving with pinhole, with visually impairing lens opacity in one or both eyes)

Need not felt	O (1)
Fear of surgery or poor result	O (2)
Cannot afford operation	O (3)
Treatment denied by provider	O (4)
Unaware that treatment is possible	O (5)
No access to treatment	O (6)
Local reason (optional)	O (7)

G. DETAILS ABOUT CATARACT OPERATION

	**Right eye**	**Left eye**
**Age at operation (years)**	☐☐	☐☐
**Place of operation**		
Government hospital	O (1)	O (1)
Voluntary / charitable hospital	O (2)	O (2)
Private hospital	O (3)	O (3)
Eye camp / improvised setting	O (4)	O (4)
Traditional setting	O (5)	O (5)
**Type of surgery**		
Non IOL	O (1)	O (1)
IOL implant	O (2)	O (2)
Couching	O (3)	O (3)
**Cost of surgery**		
Totally free	O (1)	O (1)
Partially free	O (2)	O (2)
Fully paid	O (3)	O (3)
**Cause of VA>0.3 after cataract surgery**		
Ocular comorbidity (Selection)	O (1)	O (1)
Operative complications (Surgery)	O (2)	O (2)
Refractive error (Spectacles)	O (3)	O (3)
Longterm complications (Sequelae)	O (4)	O (4)
Does not apply - can see 0.3	O (5)	O (5)
